# Homozygous *CADPS2* Mutations Cause Neurodegenerative Disease with Lewy Body‐like Pathology in Parrots

**DOI:** 10.1002/mds.29211

**Published:** 2022-09-10

**Authors:** Oswaldo Lorenzo‐Betancor, Livio Galosi, Laura Bonfili, Anna Maria Eleuteri, Valentina Cecarini, Ranieri Verin, Fabrizio Dini, Anna‐Rita Attili, Sara Berardi, Lucia Biagini, Patrizia Robino, Maria Cristina Stella, Dora Yearout, Michael O. Dorschner, Debby W. Tsuang, Giacomo Rossi, Cyrus P. Zabetian

**Affiliations:** ^1^ Veterans Affairs Puget Sound Health Care System Seattle Washington USA; ^2^ Department of Neurology University of Washington School of Medicine Seattle Washington USA; ^3^ School of Biosciences and Veterinary Medicine University of Camerino Matelica Italy; ^4^ Department of Comparative Biomedicine and Food Science University of Padova “Agripolis” Legnaro Italy; ^5^ Department of Veterinary Sciences University of Torino Torino Italy; ^6^ Department of Pathology, Center for Precision Diagnostics University of Washington Seattle Washington USA; ^7^ Department of Psychiatry University of Washington School of Medicine Seattle Washington USA

**Keywords:** *CADPS2*, Lewy body, parkinsonism, parrot, Parkinson's disease, animal model

## Abstract

**Background:**

Several genetic models that recapitulate neurodegenerative features of Parkinson's disease (PD) exist, which have been largely based on genes discovered in monogenic PD families. However, spontaneous genetic mutations have not been linked to the pathological hallmarks of PD in non‐human vertebrates.

**Objective:**

To describe the genetic and pathological findings of three Yellow‐crowned parrot (*Amazona ochrocepahala*) siblings with a severe and rapidly progressive neurological phenotype.

**Methods:**

The phenotype of the three parrots included severe ataxia, rigidity, and tremor, while their parents were phenotypically normal. Tests to identify avian viral infections and brain imaging studies were all negative. Due to their severe impairment, they were all euthanized at age 3 months and their brains underwent neuropathological examination and proteasome activity assays. Whole genome sequencing (WGS) was performed on the three affected parrots and their parents.

**Results:**

The brains of affected parrots exhibited neuronal loss, spongiosis, and widespread Lewy body‐like inclusions in many regions including the midbrain, basal ganglia, and neocortex. Proteasome activity was significantly reduced in these animals compared to a control (*P* < 0.05). WGS identified a single homozygous missense mutation (p.V559L) in a highly conserved amino acid within the pleckstrin homology (PH) domain of the calcium‐dependent secretion activator 2 (*CADPS2*) gene.

**Conclusions:**

Our data suggest that a homozygous mutation in the *CADPS2* gene causes a severe neurodegenerative phenotype with Lewy body‐like pathology in parrots. Although *CADPS2* variants have not been reported to cause PD, further investigation of the gene might provide important insights into the pathophysiology of Lewy body disorders. © 2022 The Authors. *Movement Disorders* published by Wiley Periodicals LLC on behalf of International Parkinson and Movement Disorder Society. This article has been contributed to by U.S. Government employees and their work is in the public domain in the USA.

## 1 Introduction

The development of genetic animal models that manifest the full spectrum of features in Parkinson's disease (PD) has been challenging.^1^ Currently, there are several such models, but none of them completely mimic the clinical findings and associated neuropathological hallmarks of PD.^1^ These include mice expressing truncated C‐terminus α‐synuclein which is more prone to aggregation,^2^, ^3^, ^4^ conditionally expressing either wild‐type (WT) or A53T human α‐synuclein,^5^, ^6^ and expressing A53T α‐synuclein on a null parkin^7^ or DJ‐1 background.^4^, ^8^, ^9^, ^10^, ^11^ Of the existing vertebrate models, only transgenic mice expressing A53T α‐synuclein driven by the mouse prion promoter (mPrP) display the full range of α‐synuclein pathology that is observed in human brains, which includes α‐synuclein aggregation, fibrils and truncation, α‐synuclein phosphorylation and ubiquitination, and progressive age‐dependent non‐dopaminergic neurodegeneration.^12^, ^13^, ^14^, ^15^ However, this model does not show progressive degeneration of the dopaminergic system.^1^ Conversely, autosomal recessive knockout models based on *PRKN*, *PINK1*, or *DJ‐1* genes do not show any substantial behavioral or progressive nigrostriatal pathology, nor the typical neuropathological PD hallmark of α‐synuclein aggregation.^7^, ^16^, ^17^, ^18^, ^19^, ^20^, ^21^, ^22^, ^23^

The Yellow‐crowned parrot (*Amazona ochrocephala*) is a species native to tropical Central and South America. Descriptions of neurodegenerative pathologies in parrots and other birds are uncommon, and in most instances the pathology is related to environmental exposures to toxins such as organophosphates and heavy metals that induce axonopathies.^24^ Rare cases with central nervous system involvement such as a Lafora disease‐like syndrome in cockatiels^25^ and cerebellar degeneration in parrots^26^ and chickens^27^ have been described, but Lewy body (LB) pathology has never been reported in birds. Here we present a Yellow‐crowned parrot pedigree with a severe progressive early onset neurodegenerative phenotype and widespread Lewy body‐like pathology.

## 2 Materials and Methods

### 2.1 Animals

Three 3‐month‐old Yellow‐crowned Amazons (*Amazona ochrocephala*) were brought to the School of Biosciences and Veterinary Medicine of the University of Camerino in Italy, showing severe neurological symptoms. Subsequently, DNA samples from their parents, two uncles, and their grandparents were acquired using blood collected during routine annual veterinary visits.

### 2.2 Clinical Visit and Exams

The clinical history of the three affected parrots and their family was collected. From the three affected birds, blood samples were collected at multiple times to perform hematological and biochemical analyses. Given that viral and bacterial infections can cause neurological manifestations in birds, polymerase chain reaction (PCR) testing for chlamydophila, avian polyomavirus, avian bornavirus, paramyxovirus, and beak and feather disease virus was conducted.^28^ Anti‐ganglioside antibody serology was performed to exclude any form of parrot ganglioneuritis.^28^ Brain neuroimaging was performed with a veterinary magnetic resonance imaging (MRI) 0.2 Tesla (Esaote S.p.A, Genova, Italy), using T2 (transverse and sagittal), T1 (transverse), FLAIR (dorsal), and STIR (dorsal) sequences.

### 2.3 Pathological Analysis

Given that the parrots were unable to feed themselves and the neurological symptoms worsened, with eventual death imminent, they were humanitarianly euthanized at the age of 3 months. A complete necropsy was carried out and all organs were fixed in 10% buffered formalin for histological examination. Brain tissue was stained with hematoxylin and eosin, Congo red dye, Luxol fast blue, PAS, and immunohistochemistry (IHC) was performed using an anti‐synaptophysin antibody (Agilent Technologies, Inc., Santa Clara, CA, USA), anti‐neurofilament antibody (Merck KGaA, Darmstadt, Germany), and an α‐synuclein antibody (Santa Cruz Biotechnology, Inc., Dallas, TX, USA). Small portions of brain (1 mm^3^) were fixed in 2.5% glutaraldehyde for 24 hours and then in Millonig buffer for electron microscopy. After dehydration, the sections were embedded in epoxy resin. Semi‐thin toluidine blue 1% stained sections were produced to assess target areas for ultrastructural analysis. Ultra‐thin sections (75 nm) were then mounted on copper grids and examined under a Philips EM208S (FEI UK, Cambridge, UK) transmission electron microscope.

To explore the involvement of a *CADPS2* mutation in the pathology, IHC was performed with a CADPS2 polyclonal antibody (Invitrogen Corporation, MA, USA) followed by a TUNEL assay. The expression of CADPS2 was analyzed and quantified using ImageJ/Fiji 1.52p software (NIH, USA) as previously reported.^29^ As a control for IHC, western blot, and proteasomal analysis, the brain of a healthy parrot of the same species that died without brain lesions was used.

### 2.4 Western Blot and Proteasomal Analysis

Brains were homogenized in 50 mM Tris buffer, 150 mM KCl, 2 mM EDTA, pH 7.5 (1:5 weight/volume of buffer). Homogenates were immediately centrifuged at 13,000*g* for 20 minutes at 4°C and the supernatant was collected for enzyme activity assays and western blotting. Protein content was determined by the Bradford method^30^ using bovine serum albumin (BSA) as standard.

Proteasome peptidase activities in brain homogenates were determined using synthetic fluorogenic peptides: Suc‐Leu‐Leu‐Val‐Tyr‐AMC was used for chymotrypsin‐like (ChT‐L) activity, Z‐Leu‐Ser‐Thr‐Arg‐AMC for trypsin‐like (T‐L) activity, and Z‐Leu‐Leu‐Glu‐AMC for peptidyl‐glutamyl‐peptide hydrolyzing (PGPH) activity.^31^ The incubation mixture contained brain homogenates (15 μg total proteins), the proper substrate (5 μM final concentration), and 50 mM Tris–HCl pH 8.0, up to a final volume of 100 μL. Incubation was performed at 37°C for 60 minutes and the fluorescence of the hydrolyzed 7‐amino‐4‐methyl‐coumarin (AMC) was detected (AMC, λexc = 365 nm, λem = 449 nm; pAB, λexc = 304 nm, λem = 664 nm) on a SpectraMax Gemini XPS microplate reader. The 26S proteasome ChT‐L activity was tested by including in the reaction mix 10 mM MgCl_2_, 1 mM dithiothreitol, and 2 mM ATP. Brain homogenates were also analyzed through western blotting assays using anti α‐synuclein (C‐20) primary antibody (sc‐7011, from Santa Cruz Biotechnology, Heidelberg, Germany). The bands were quantified by using a densitometric algorithm. Each western blot was scanned (16 bits greyscale) and the obtained digital data were processed through Image J (NIH)^32^ to calculate the background mean value and its standard deviation. The background‐free image was then obtained by subtracting the background intensity mean value from the original digital data. The integrated densitometric value associated with each band was then calculated as the sum of the density values over all the pixels belonging to the considered band having a density value higher than the background standard deviation. The band densitometric value was then normalized to the relative GAPDH signal intensity. The ratios of band intensities were calculated within the same western blot. All calculations were carried out using the Matlab environment (The MathWorks Inc., MA, USA).^33^

### 2.5 Whole Genome Sequencing Methods

**FIG 1:**
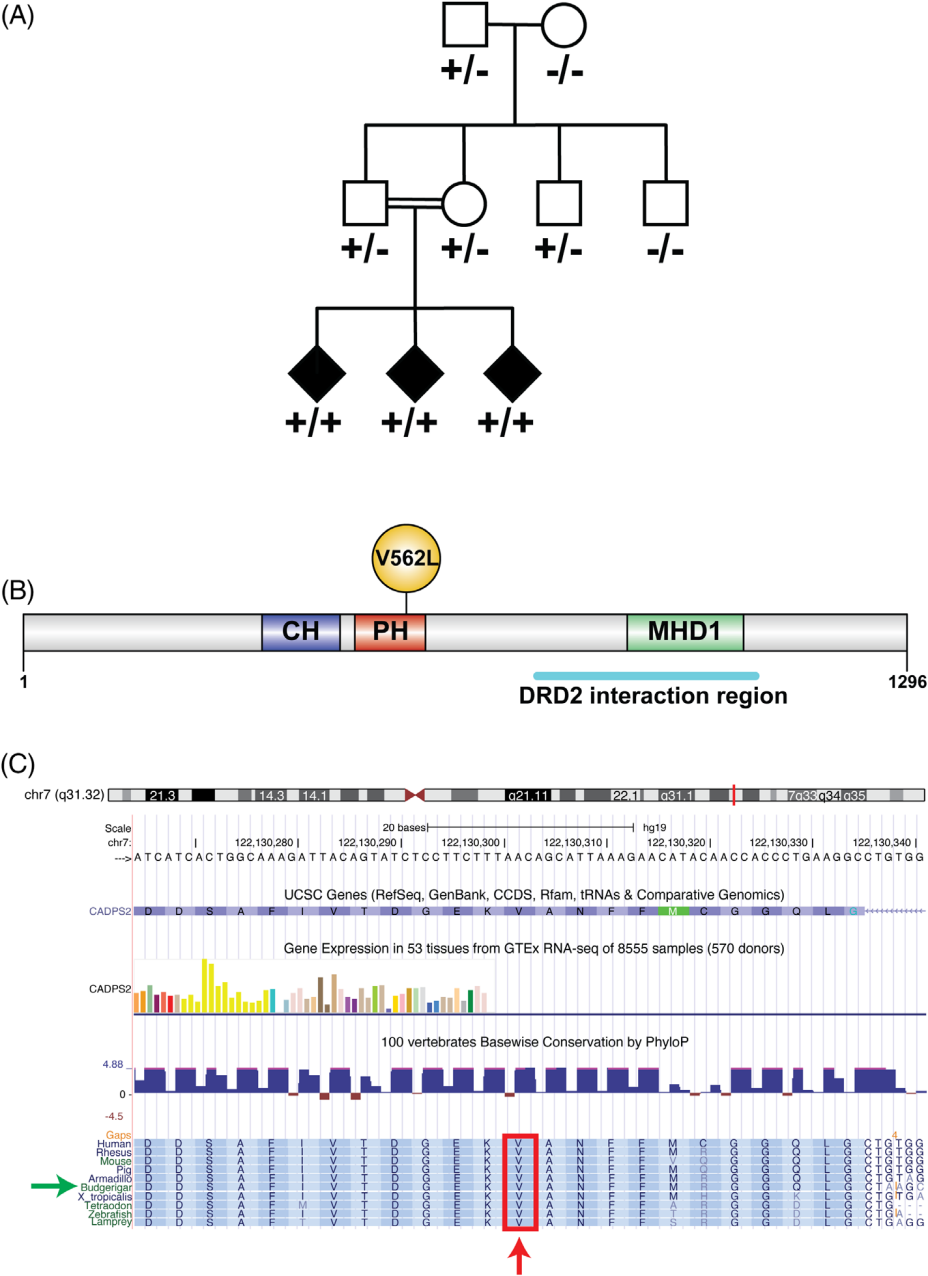
(A) Parrot pedigree. p.V559L segregated with disease in a recessive pattern. The grandparents were captured in the wild, but the remainder of the parrots were born in captivity. Consanguinity of both parents is represented by a horizontal double line. Affected parrots are represented with black symbols and unaffected parrots with clear symbols. Plus and minus symbols indicate mutant and wild‐type alleles, respectively. (B) Human CADPS2 protein structure. CH domain, calponin homology domain (family of actin‐binding domains found in both cytoskeletal proteins and signal transduction proteins); PH domain, the pleckstrin homology domain is a protein domain that occurs in a wide range of proteins involved in intracellular signaling or as constituents of the cytoskeleton; MHD1 domain, the munc13‐homology domain 1 may function in a Munc13‐like manner to regulate membrane trafficking; DRD2 (dopamine receptor D2) interaction region; (C) amino acid V562 schematic view that shows its high conservation across species. Red arrow shows the affected valine. Green arrow indicates the budgerigar (common parakeet) which was used as the reference sequence for the parrot.

DNA was extracted from liver of the three affected parrots and from blood of their family members. Whole genome sequencing (WGS) of the three offspring and their parents was performed at the University of Washington with 1 μg of DNA on a HiSeq 2000 Sequencing System (Illumina, San Diego, CA). The WGS data were aligned using a standard BWA pipeline to the budgerigar (*Melopsittacus undulatus*) reference genome (Budgerigar v6.3)^34^ which contains 25,212 scaffolds of an undetermined number of chromosomes. The budgerigar genome shares more than 99.9% homology with the *Amazona ochrocephala* genome. Annotation was performed with ANNOVAR software^35^ using Ensembl and UCSC gene databases for the Budgerigar v6.3 reference genome. Given the recessive inheritance pattern of the disease (both parents were healthy and related to each other with their three offspring affected), all intergenic variants were first removed, and the remaining variants were restricted to those for which both parents were heterozygous, and the three offspring were homozygotes. The ortholog variants in humans were annotated according to the gene transcript ENST00000449022.7. The candidate variant identified in the WGS analysis was assessed for co‐segregation with disease by Sanger sequencing in all members of the pedigree (Fig. 1A). Primers were designed using Primer3 v.0.4.0 (https://bioinfo.ut.ee/primer3-0.4.0/) and are available on request.

## 3 Results

### 3.1 Clinical Description

**FIG 2:**
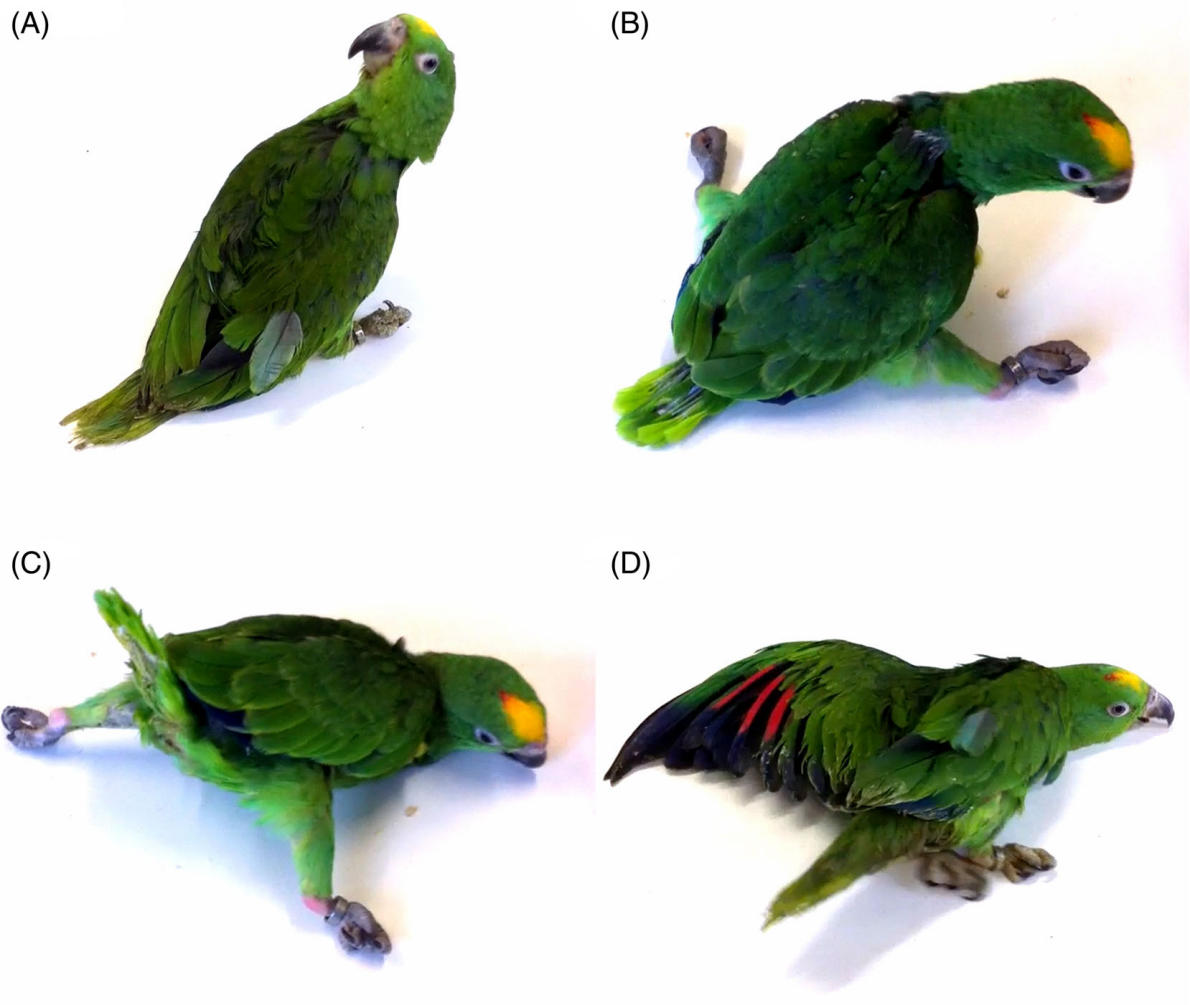
Clinical features. The affected parrots showed a twisted head (A), arthrogryposis (B), and impaired balance and coordination leading to falls and an inability to maintain an upright position (C, D).

A severe neurological condition was observed in three hand‐reared parrot siblings birthed from two different clutches from the same parents, who were siblings (Fig. 1A). The grandparents were wild parrots, while the rest of the animals had been born in captivity. The three affected birds hatched from artificially incubated eggs and were hand‐fed. On veterinary examination they exhibited uncoordinated movements, head tilt, and stargazing (twisted back; see Supplementary video and Fig. 2A–D) early in life (2 months). The breeder reported that they never exhibited normal behavior or movements since birth and were never able to assume a physiological position in the container where they were housed. The early motor signs were stiff neck muscles, and often hyperextension of the limbs, in association with early‐onset persistent tremor. Tremor was the most noticeable sign observed. It usually began intermittently in one wing and increased considerably when the parrots were under stress or fatigued. The tremor rapidly became bilateral and diffuse, though some degree of asymmetry was still evident. The parrots were unable to perch and they had to be hand‐fed despite their age, as they were not able to eat independently. One of the three siblings developed aspiration pneumonia due to his inability to assume an upright position.

### 3.2 Complementary Analyses

Hematological and biochemical findings from blood samples obtained at multiple times were not diagnostic. PCR testing for avian polyomavirus, avian bornavirus, paramyxovirus, beak and feather disease virus, and chlamydophila were all negative. The parrots were also seronegative for anti‐ganglioside antibodies. Brain MRI was negative for notable pathology.

**FIG 3:**
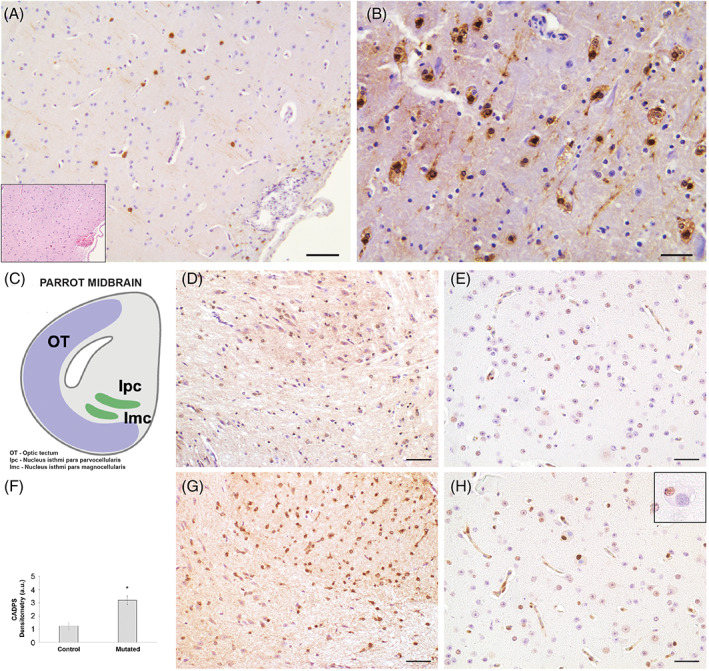
(A, B). Mutated parrot midbrain and periventricular area. (A) In the periventricular area some intracytoplasmic inclusions in neurons are immunopositive for α‐synuclein (brown staining). In the insert, the same hematoxylin and eosin (H&E)‐stained section indicates the submeningeal periventricular area in which a scattered inflammatory infiltrate is present. (B) Higher magnification of the hippocampal area showing that the cytoplasm of a large proportion of neurons stained positive for α‐synuclein. A vacuolated appearance of the cytoplasm in these neurons is apparent. Immunosections were counterstained with Meyer's hematoxylin. Scale bar A = 250 μm, B = 150 μm. (C–H) Mutated and control parrot midbrain comparison. (C) Schematic diagram of the bird midbrain. Modified from Krauzlis et al.^58^ with permission of Elsevier Science & Technology Journals. OT, optic tectum; lpc, nucleus isthmi pars parvocellularis; lmc, nucleus isthmi pars magnocellularis. (D) Presence of anti‐CADPS2 antibody stain in the periventricular area of the midbrain in a healthy control parrot (D) compared to an affected parrot (G). Note the difference of expression and localization, as also quantified in (F), in the CADPS2‐positive neurons (brown stain). (F) CADPS2 expression analyzed and quantified using ImageJ/Fiji 1.52p software (NIH, USA). The data point marked with an asterisk is statistically significant compared to the affected bird (*P < 0.05). (E, H) Presence of neurons showing TUNEL‐positive nuclei in the same parrot's midbrain region. There is a high concentration of TUNEL‐positive, brown‐stained neuronal nuclei in a clear pre‐apoptotic state in the section belonging to the affected parrot (H), while the TUNEL‐positive nuclei in the same area of the healthy parrot are few and very lightly stained (E). Immunosections were counterstained with Meyer's hematoxylin. Scale bar D and G = 250 μm; E and H = 200 μm.

On pathological examination, the main microscopic findings observed in the brain included moderate to severe neuronal loss, microspongiosis, and reactive astrogliosis. There were widely distributed variably sized (up to ~100 μm in diameter), round to elongate, well‐defined eosinophilic structures that occasionally contained fine boat‐shaped clefts, imparting a crinkled appearance in both gray and white matter. These abundant neuronal and axonal eosinophilic inclusions lacked a distinctive core and halo and resembled Lewy bodies (LBs) and Lewy neurites,^36^, ^37^ which did not stain positively with Congo red, Luxol fast blue, or PAS. Large spherical bodies were occasionally observed in cerebellar Purkinje cells, and multiple small round bodies composed of similar material were noted within pericardial and proventricular ganglia. IHC performed with anti‐synaptophysin and neurofilament antibodies failed to stain the eosinophilic bodies. In contrast, strong α‐synuclein immunostaining suggested that the round intraneuronal structures were consistent with cortical Lewy body‐like inclusions (LBLIs) (Fig. 3). These LBLIs were also present in the neocortex, amygdala, hypothalamus, periaqueductal gray matter, dorsal vagal nucleus, in some cerebellar Purkinje cells, and in the basal ganglia. Some pale bodies and axonal spheroids were present in the same structures. There were no plaques, tangles, or granulovacuolar degeneration in the hippocampal formation. The IHC against α‐synuclein confirmed the presence of LBLIs in the above‐mentioned structures. The CADPS2 immunostaining revealed a homogeneously distributed increase of the CADPS2 signal in the affected parrots' brains when compared to a control (2.5‐fold higher than control; Fig. 3F), but LBLIs did not stain positive for CADPS2 (Fig. 3G).

In the affected parrots, a conspicuous feature of neurons harboring α‐synuclein‐positive LBLIs was somal chromatolytic changes, defined by distension of the cell body, displacement of the nucleus toward the periphery of the soma, and dissolution of the Nissl substance. These neurons also showed nuclear condensation. Subsets of large neurons had this chromatolytic signature, showing TUNEL‐positive staining (Fig. 3H). Subsets of neurons in brainstem and neocortex were TUNEL‐positive, indicating cells with double‐stranded DNA breaks. In apoptotic neurons, varying degrees of nuclear alterations, ranging from moderate to major chromatin condensation and, in some of these neurons, disappearance of the nucleolus, were observed. Though the nuclear envelope appeared grossly intact it was convoluted. Some of these dying neurons were partially or totally engulfed by glial cells, suggesting an ongoing phagocytic process.

**FIG 4:**
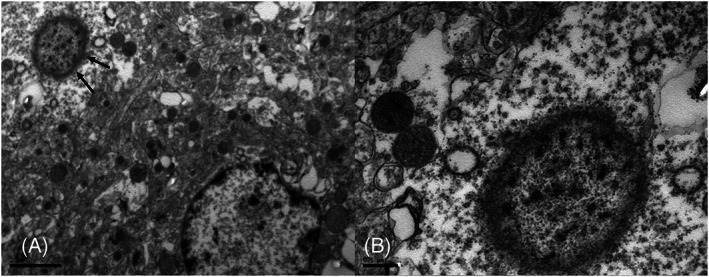
Transmission electron microscopy of a mutated parrot neuron. (A) Intracytoplasmic Lewy body‐like inclusions (LBLI) (black arrows) characterized by accumulations of short granular electrondense material on a translucid background and organized in short filaments at the periphery. (B) Higher magnification of the same LBLI. A general reduction of mitochondria is observed in the neuron, and the two mitochondria near the LBLI show loss of cristae and a rounded‐degenerate appearance. In neurons containing LBLIs, many mitochondria displayed spherical pleiomorphism with poorly defined and irregular cristae and a finely granular matrix. Some small spherical bodies were also observed without cristae. Scale bar A = 2 μm; B = 0.5 μm.

Finally, electron microscopy imaging identified the eosinophilic material constituting LBLIs as accumulations of short granular electrondense material on a translucid background and organized in short filaments at the periphery (Fig. 4). The presence of an electrondense core, characteristic of LBs in neurons of patients with PD, was not evident in our samples. The material was largely confined to neuronal bodies, and accumulation within the axons was minimal. In LBLIs‐containing neurons, the mitochondria were reduced in number and had a dysmorphic appearance with vacuolization and poorly defined and irregular cristae (Fig. 4).

### 3.3 Proteasome and Western Blot Analyses

**FIG 5:**
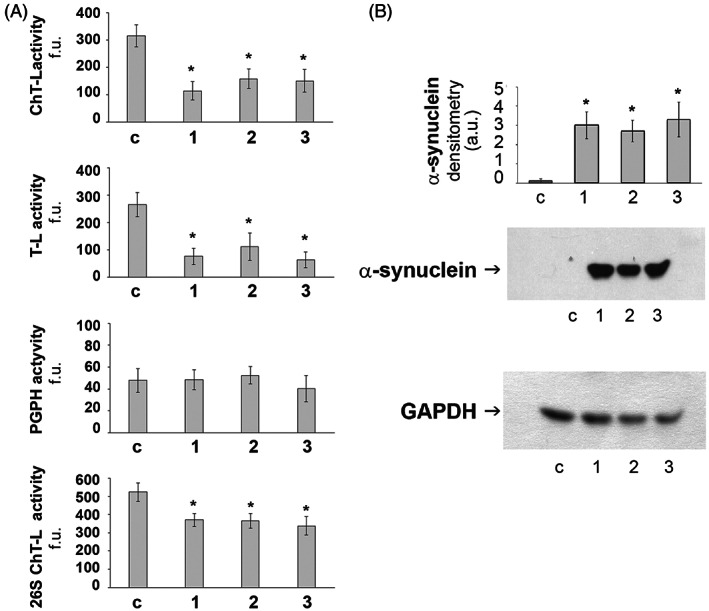
Proteasome activity and α‐synuclein levels. (A) Proteasome activity in control (c) and affected (1–3) parrots. 20S proteasome chymotrypsin‐like (ChT‐L), trypsin‐like (T‐L), and peptidyl‐glutamyl‐peptide hydrolyzing (PGPH) activities and the 26S proteasome ChT‐L activity were measured in brain homogenates as described in the Materials and Methods section. Results are expressed as fluorescence units (f.u.). Data points marked with an asterisk are statistically significant compared to the control bird (*P < 0.05). (B) Western blot detection of α‐synuclein levels in activity in brain homogenates of control (c) and affected (1–3) parrots. The densitometric analyses obtained from five separate blots and representative immunoblots are shown. Anti‐GAPDH antibody was used to confirm equal protein loading and to normalize the target protein. The detection was performed using an ECL western blotting analysis system. Data points marked with an asterisk are statistically significant compared the control bird (*P < 0.05).

Significantly reduced ChT‐L and T‐L proteasomal activities were observed in affected parrots compared to the control (*P* < 0.05; Fig. 5A), suggesting a deficit in proteostasis. Moreover, the densitometric analyses obtained from five separate blots detected a significant increase of α‐synuclein levels in brain homogenates of each of the three parrots compared to the control (*P* < 0.05; Fig. 5B).

### 3.4 Whole Genome Sequencing Results

We identified 88 variants genome‐wide that remained after filtering was performed (see Supplementary Table S1). Twelve were upstream variants, six were downstream variants, one was located in the 3′ UTR region of a gene, 62 were intronic variants, five were synonymous variants, and one was a missense variant that was located in the calcium‐dependent secretion activator 2 (*CADPS2*; JH556570.1:3828757C > G; c.1675G > C; p.V559L) gene. The ortholog position and mutation in the human *CADPS2* gene located on chromosome 7 is: g.122130303C > G; c.1684G > C; p.V562L. This amino acid is highly conserved across species, including invertebrates (see Fig. 1B, 1C), and resides in the pleckstrin homology (PH) domain of the protein.

## 4 Discussion

In this study we report three parrot siblings with a rapidly progressive neurodegenerative disease which we propose is caused by a spontaneous homozygous missense mutation (c.1675G > C; p.V559L) in the *CADPS2* gene. The affected parrots displayed some clinical features of PD including rigidity and tremor though their phenotype was not limited to pure parkinsonism. Their brains displayed widespread neuronal loss and intraneuronal α‐synuclein and ubiquitin‐positive inclusions consistent with LBLIs. The affected neurons displayed dysmorphic mitochondria with morphological similarities to the shrunken, swollen, or vacuolated mitochondria that have been reported in the brains of some PD patients.^38^ Many of the LBLIs‐containing neurons were TUNEL‐positive indicating an apoptotic state.

The onset of disease in these parrots was very early, even in comparison to early‐onset monogenic forms of PD in humans. This species of parrot has a long lifespan and has been reported to live up to 56 years in captivity.^39^ The fact that the affected parrots had abnormal movements since birth and displayed widespread LBLIs throughout the brain by age 3 months suggests that abnormal α‐synuclein aggregation began during embryonic stages.

The *CADPS2* gene codes for a member of the CADPS protein family.^40^ This family includes two main genes, *CADPS1* and *CADPS2*, which are expressed at the highest levels in brain.^41^, ^42^, ^43^ A mouse study showed that CADPS1 regulated catecholamine release from neuroendocrine cells, while CADPS2 regulated the release of two neurotrophins, brain‐derived neurotrophic factor (BDNF) and neurotrophin‐3 (NT3).^44^ A more recent study of CAPDS2 distribution in mouse brain reported the highest concentrations of CADPS2 immunoreactivity in the midbrain, cerebellum, and hippocampus. Within the midbrain, peak immunostaining was observed in the cells and mesh‐like fiber system of the substantia nigra (SN), ventral tegmental area (VTA), and interpeduncular nucleus (IPN), and overlapped with tyrosine hydroxylase (TH; a dopaminergic neuron marker^45^) immunoreactivity in the SN and VTA but not in the IPN. Clear immunoreactivity for CADPS2, but not CADPS1, was substantially localized to TH‐positive neurons in mesencephalic‐striatal co‐cultures.^46^ This suggested that CADPS2 is predominantly expressed in dopaminergic neurons of the SN and VTA.^46^ In addition, the same study showed that CADPS2 immunostaining overlapped with BDNF–TrkB signaling in the hippocampus.^46^

Taken together, these data suggest that CADPS2 regulates presynaptic BDNF release in dopaminergic neurons, which is particularly relevant to PD and other neurodegenerative diseases. In animal models of PD, BDNF enhances the survival of dopaminergic neurons, improves dopaminergic neurotransmission, and accelerates recovery of motor function.^47^ The mutation that we observed in this study (p.V559L) occurs in the PH domain of the protein and previous in vitro experiments comparing different mouse splice isoforms suggest that this region is required for efficient BDNF release.^48^ Thus, one mechanism by which mutations in *CADPS2* might induce neurodegeneration is by altering the appropriate release of BDNF.

*CADPS2* is located in the “autism susceptibility locus 1” on chromosome 7q31.32^49^ and genetic variants have been associated with autism spectrum disorders and intellectual disability.^50^ A Cadps2^−/−^ knockout mouse was generated and assessed for behaviors that model facets of autistism.^48^ The mice displayed several “autistic‐like behavioral phenotypes” including impaired social interactions, hyperactivity, and decreased exploratory behavior, but they did not show any deficits in motor function and had normal lifespans and reproductive ability. Their brains exhibited several abnormal cell phenotypes, such as significantly fewer parvalbumin‐positive GABAergic interneurons in neocortex and hippocampus, but they did not display the widespread neuronal loss observed in the parrots we report here. While no LBLIs were reported, α‐synuclein immunohistochemistry was not performed so it is unclear whether abnormal α‐synuclein aggregates were present. There are several potential explanations for the differences in phenotype between these Cadps2^−/−^ knockout mice and the homozygous p.V559L parrots including large differences in the genetic background of the organisms and the nature of the specific mutations. While no CADPS2 protein was detected in the Cadps2^−/−^ mouse brains, 2.5‐fold elevated levels of the mutant protein were found in the brains of the parrots, and how p.V559L alters CADPS2 function is not yet known. An example of such phenotypic heterogeneity has been reported for *RAB39B* in humans. Null mutations in the gene often result in mental retardation associated with autism and/or epilepsy,^51^ while a missense mutation in the gene (p.G192R) causes typical levodopa‐responsive PD.^52^

Two of the most important familial PD‐related genes are *LRRK2* and *SNCA*.^53^ Interestingly, one study performed in cell lines with overexpression of either LRRK2 or α‐synuclein showed that CADPS2 expression is differentially regulated by LRRK2 and SNCA.^54^ This study showed a significant upregulation (~2‐fold) in both WT and G2019S‐LRRK2‐expressing cells, when compared to control SHSY5Y cells. Therefore, overactivation of LRRK2, independent of the G2019S mutation, led to increased CADPS2 transcriptional activation suggesting that enhanced LRRK2 cellular function would be sufficient to induce transcriptional dysregulation.^54^ The same study evaluated CADPS2 promoter‐dependent transcriptional activity in human neuroblastoma SK‐N‐SH cells overexpressing WT or the PD‐causing *SNCA* A30P mutation. In contrast to LRRK2‐overexpressing cells, CADPS activity was reduced ~20% and ~ 35% in WT‐ and A30P cells, respectively, when compared to control cells. The effect was again independent from disease‐causing mutations.^54^ Transcriptomic analyses in mice overexpressing human WT α‐synuclein have suggested that SNCA may control synaptic vesicle release by downregulating the expression of *Cadps2*,^55^ which is critical for constitutive vesicle trafficking and secretion.^56^ Thus, CADPS2 expression might be regulated in part by genes that are well‐established as playing a role in PD pathogenesis.

A recent study that analyzed the contribution of all midbrain cell types to PD pathology using single‐cell sequencing of human mesencephalon tissue identified a neuronal cell cluster characterized by CADPS2 overexpression and low TH levels that was almost exclusively present in idiopathic PD but not controls.^57^ Aside from low TH these neurons displayed a pattern of neuronal markers similar to typical dopamine neurons. Thus, the authors suggested that these high CADPS2‐expressing cells might represent degenerating dopamine neurons that have lost their dopaminergic identity.^57^

Our results suggest that mutations in the *CADPS2* gene cause a severe neurodegenerative phenotype associated with LBLIs in parrots. Although *CADPS2* variants have not been reported to cause neurodegenerative diseases in humans, further investigation of the gene in animal models might provide important insights into the pathophysiology of LB disorders.

## Author Roles

O.L.‐B.: conceptualization, methodology, data curation, formal analysis, visualization, writing – original draft preparation, review and editing. L.G.: resources, methodology, investigation, writing – original draft preparation. L.B., A.M.E., V.C., R.V., F.D., A.‐R.A., S.B., Lu.B., P.R., and M.C.S.: methodology, investigation, writing – review & editing. D.Y.: project administration, validation, writing – review and editing. M.O.D.: investigation, data curation, writing – review and editing. D.W.T.: conceptualization, writing – review and editing and supervision. G.R.: resources, methodology, investigation, writing – original draft preparation and supervision. C.P.Z.: conceptualization, funding acquisition, writing – original draft preparation, and supervision.

## Financial Disclosures

None of the authors report any competing financial interests for this work.

## Supporting information


**Table S1.** Mutations identified in the Amazon parrots that met selection criteria.

**Video S1.** Supplementary Video.

## Data Availability

The data that support the findings of this study are available from the corresponding authors upon request.
