# Supplementary material for: Homozygous CADPS2 Mutations Cause Neurodegenerative Disease with Lewy Body‐like Pathology in Parrots
Source: Mov Disord. 2022 Sep 10;37(12):2345–54. doi: 10.1002/mds.29211 (PMC9772200; doi:10.1002/mds.29211)
Supplement: Supplementary file 1 — Table S1. Mutations identified in the Amazon parrots that met selection criteria. [file MDS-37-2345-s001.docx]

Homozygous *CADPS2* mutations cause neurodegenerative disease with Lewy body-like pathology in parrots – Supplementary Material

Oswaldo Lorenzo-Betancor^1,2,*^, Livio Galosi^3,*^, Laura Bonfili^3^, Anna Maria Eleuteri^3^, Valentina Cecarini^3^, Ranieri Verin^4^, Fabrizio Dini^3^, Anna-Rita Attili^3^, Sara Berardi^3^, Lucia Biagini^3^, Patrizia Robino^5^, Maria Cristina Stella^5^, Dora Yearout^1^, Michael O. Dorschner^6^, Debby W. Tsuang^2,7,†^, Giacomo Rossi^3,†^, Cyrus P. Zabetian^1,2,†^

^1^ Veterans Affairs Puget Sound Health Care System, Seattle, WA, USA

^2^ Department of Neurology, University of Washington School of Medicine, Seattle, WA, USA

^3^ School of Biosciences and Veterinary Medicine, University of Camerino, Matelica, Italy

^4^ Department of Comparative Biomedicine and Food Science, University of Padova AGRIPOLIS, Legnaro, Italy

^5^ Department of Veterinary Sciences, University of Torino, Torino, Italy

^6^ Department of Pathology, Center for Precision Diagnostics, University of Washington, Seattle, WA 98195, USA.

^7^ Department of Psychiatry, University of Washington School of Medicine, Seattle, WA, USA

^*^Both authors contributed equally to this paper

^†^ Co-corresponding authors

**Supplementary table 1. Mutations identified in the Amazon parrots that met selection criteria**

| **Gene ID** | **Mutation** | **Human ortholog** | **Parrots gene name** |
| --- | --- | --- | --- |
| **Upstream variants** | | | |
| *ENSMUNG00000007860* | JH556440:1456511 T>G | No orthologs | Not characterized |
| *CCDC3* | JH556467:854099 G>T | *CCDC3* | Coiled-coil domain containing 3 |
| *ENSMUNG00000011465* | JH556513:37413 G>T | No orthologs | Not characterized |
| *ARHGAP29* | JH556574:3047604 G>A | *ARHGAP29* | Rho GTPase activating protein 29 |
| *HES1* | JH556581:130341 T>C | *HES1* | Hes family bHLH transcription factor 1 |
| *SEMA3A* | JH556593:904056 A>G | *SEMA3A* | Semaphorin 3A |
| *MSI2* | JH556627:6313140 AG>A | *MSI2* | Musashi RNA binding protein 2 |
| *MSI2* | JH556627:6313144 T>C | *MSI2* | Musashi RNA binding protein 2 |
| *MSI2* | JH556627:6313148 TCC>T | *MSI2* | Musashi RNA binding protein 2 |
| *MSI2* | JH556627:6313152 TAG>T | *MSI2* | Musashi RNA binding protein 2 |
| *MSI2* | JH556627:6313155 A>T | *MSI2* | Musashi RNA binding protein 2 |
| *MSI2* | JH556627:6313158G C>G | *MSI2* | Musashi RNA binding protein 2 |
| **Downstream variants** | | | |
| *GOLGB1* | JH556230:144106 T>C | *GOLGB1* | Golgin B1 |
| *SELENOF* | JH556574:2880439 A>G | *SELENOF* | Selenoprotein F |
| *KNG1* | JH556579:7170399 G>T | *KNG1* | Kininogen 1 |
| *POMT2* | JH556597:2370780 A>G | *POMT2* | Protein O-mannosyltransferase 2 |
| *CPNE3* | JH556607:8816919 T>A | *CPNE3* | Copine 3 |
| *KCNK12* | JH556611:3308002 A>G | *KCNK12* | Potassium two pore domain channel subfamily K member 12 |
| **3’ UTR region** | | | |
| *ENSMUNG00000009678* | JH556472:111791T>C | No orthologs | Dual specificity testis-specific protein kinase 1-like |
| **Intronic variants** | | | |
| *HPS3* | JH556131:245809 T>C | *HPS3* | HPS3 biogenesis of lysosomal organelles complex 2 subunit 1 |
| *RNF141* | JH556236:326470 T>G | *RNF141* | Ring finger protein 141 |
| *EFTUD2* | JH556240:20215 T>C | *EFTUD2* | Elongation factor Tu GTP binding domain containing 2 |
| *RNF123* | JH556294:363891 G>T | *RNF123* | Ring finger protein 123 |
| *CACNA2D2* | JH556294:1600415 T>G | *CACNA2D2* | Ca2^+^ voltage-gated channel auxiliary subunit alpha 2 delta 2 |
| *EIF2B5* | JH556422:124552 T>C | *EIF2B5* | Eukaryotic translation initiation factor 2B subunit epsilon |
| *SFXN1* | JH556443:1749967 C>T | *SFXN1* | Sideroflexin 1 |
| *PPM1E* | JH556455:912740 T>C | *PPM1E* | Protein phosphatase, Mg2^+^/Mn^2^+ dependent 1E |
| *PPP2R3A* | JH556459:721355 G>C | *PPP2R3A* | Protein phosphatase 2 regulatory subunit B, alpha |
| *EPHB1* | JH556459:1214019 G>T | *EPHB1* | EPH receptor B1 |
| *RYK* | JH556459:2115989 A>G | *RYK* | Receptor like tyrosine kinase |
| *CCDC3* | JH556467:811114 G>A | *CCDC3* | Coiled-coil domain containing 3 |
| *OPTN* | JH556467:878281 G>A | *OPTN* | Optineurin |
| *ENSMUNG00000015055* | JH556467:1062317 G>C | No orthologs | pre-mRNA processing factor 18 |
| *DMD** | JH556496:339158 T>C* | *DMD* | Dystrophin |
| *MGMT* | JH556537:1071186 G>A | *MGMT* | O-6-methylguanine-DNA methyltransferase |
| *LTBP1* | JH556557:3812706 C>T | *LTBP1* | Latent transforming growth factor beta binding protein 1 |
| *BRINP1* | JH556563:892106 T>G | *BRINP1* | BMP/retinoic acid inducible neural specific 1 |
| *FANCD2* | JH556566:2038768 A>G | *FANCD2* | FA complementation group D2 |
| *CNST* | JH556569:6571419 A>G | *CNST* | Consortin, connexin sorting protein |
| *MET* | JH556570:1320112 T>C | *MET* | MET proto-oncogene, receptor tyrosine kinase |
| *CPED1* | JH556570:3154117 T>A | *CPED1* | Cadherin like and PC-esterase domain containing 1 |
| *CADPS2* | JH556570:3827987 G>A | *CADPS2* | Calcium dependent secretion activator 2 |
| *GRM8* | JH556570:5483008 T>C | *GRM8* | Glutamate metabotropic receptor 8 |
| *GRM8* | JH556570:5505143 C>T | *GRM8* | Glutamate metabotropic receptor 8 |
| *GRM8* | JH556570:5505154 G>A | *GRM8* | Glutamate metabotropic receptor 8 |
| *GRM8* | JH556570:5711450 G>T | *GRM8* | Glutamate metabotropic receptor 8 |
| *UTS2B* | JH556574:1593897 T>C | *UTS2B* | Urotensin 2B |
| *GOLIM4* | JH556579:1719630 T>C | *GOLIM4* | Golgi integral membrane protein 4 |
| *NAALADL2* | JH556579:4533435 A>G | *NAALADL2* | N-acetylated alpha-linked acidic dipeptidase like 2 |
| *VOPP1* | JH556580:1511178 C>T | *VOPP1* | WW domain binding protein |
| *ADAMTS2* | JH556582:2582462 G>A | *ADAMTS2* | ADAM metallopeptidase with thrombospondin type 1 motif 2 |
| *PLCB1* | JH556583:7325538 A>T | *PLCB1* | Phospholipase C beta 1 |
| *YDJC* | JH556586:4414982 A>G | *YDJC* | YdjC chitooligosaccharide deacetylase homolog |
| *CAMSAP2* | JH556589:5850269 T>C | *CAMSAP2* | Calmodulin regulated spectrin assoc protein fam member 2 |
| *SEMA3A* | JH556593:981396 A>G | *SEMA3A* | Semaphorin 3A |
| *NAPEPLD* | JH556593:4793469 T>C | *NAPEPLD* | N-acyl phosphatidylethanolamine phospholipase D |
| *PUS7* | JH556593:5909299 C>T | *PUS7* | Pseudouridine synthase 7 |
| *PRKAR2B* | JH556593:6494204 A>G | *PRKAR2B* | Protein kinase cAMP-dependent type II regulatory subunit beta |
| *CD151* | JH556599:2918486 C>T | *CD151* | CD151 molecule (Raph blood group) |
| *NOVA1* | JH556599:9765500 C>G | *NOVA1* | NOVA alternative splicing regulator 1 |
| *EGLN3* | JH556599:12934752 A>G | *EGLN3* | Egl-9 family hypoxia inducible factor 3 |
| *ENSMUNG00000015432* | JH556600:9258645 A>G | No orthologs | Forkhead box P1 |
| *SUMF1* | JH556600:12184762 T>C | *SUMF1* | Sulfatase modifying factor 1 |
| *ENSMUNG00000014258* | JH556605:2875840 C>T | No orthologs | Guanylate cyclase soluble subunit beta-2-like |
| *LARGE1* | JH556605:13276382 G>A | *LARGE1* | LARGE xylosyl- and glucuronyltransferase 1 |
| *RDH10* | JH556607:3245659 T>G | *RDH10* | Retinol dehydrogenase 10 |
| *SDC2* | JH556607:12770380 G>A | *SDC2* | Syndecan 2 |
| *SLC44A5* | JH556608:18578303 T>C | *SLC44A5* | Solute carrier family 44 member 5 |
| *CDC42BPA* | JH556611:4757071 C>T | *CDC42BPA* | CDC42 binding protein kinase alpha |
| *TGFB2* | JH556611:7687237 A>G | *TGFB2* | Transforming growth factor beta 2 |
| *ENSMUNG00000000832* | JH556614:7806740 T>C | No orthologs | Not characterized |
| *SCUBE2* | JH556615:23817043 G>A | *SCUBE2* | Signal peptide, CUB domain and EGF like domain containing 2 |
| *GTF2H1* | JH556615:27192731 A>G | *GTF2H1* | General transcription factor IIH subunit 1 |
| *ENSMUNG00000013397* | JH556617:30408533 G>C | No orthologs | Unconventional myosin-X-like |
| *PDLIM1* | JH556617:31042012 C>T | *PDLIM1* | PDZ and LIM domain 1 |
| *TGFBR3* | JH556617:34306232 C>T | *TGFBR3* | Transforming growth factor beta receptor 3 |
| *SPSB4* | JH556617:35184609 A>G | *SPSB4* | SplA/ryanodine receptor domain and SOCS box containing 4 |
| *ENSMUNG00000014151* | JH556617:37077077 A>G | No orthologs | Glypican-5-like |
| *LIMS2* | JH556617:39010271 T>G | *LIMS2* | LIM zinc finger domain containing 2 |
| *DPYD* | JH556617:39665970 A>C | *DPYD* | Dihydropyrimidine dehydrogenase |
| *VTI1A* | JH556628:6394349 C>T | *VTI1A* | Vesicle transport through interaction with t-SNAREs 1A |
| **Synonymous variants** | | | |
| *SPEN* | JH556559:383406 G>T | *SPEN* | Spen family transcriptional repressor |
| *POT1* | JH556570:4837014 T>C | *POT1* | Protection of telomeres 1 |
| *ENSMUNG00000011074* | JH556581:464702 C>T | No orthologs | Protein phosphatase 1 regulatory inhibitor subunit 2 |
| *MYOM1* | JH556616:28872447 G>A | *MYOM1* | Myomesin 1 |
| *ENSMUNG00000008400* | JH556617:7477768 A>T | Not orthologs | Tet methylcytosine dioxygenase 1 |
| **Missense variant** | | | |
| ***CADPS2*** | **JH556570:3828757 C>G** | ***CADPS2*** | **Calcium dependent secretion activator 2** |

* The ortholog position in human *DMD* gene is intronic, too.
